# 

**Published:** 1874-07

**Authors:** 


### ACKNOWLEDGMENTS.

Atlanta Daily Herald; Rome Daily Commercial; American Journal of Pharmacy; American Practitioner; Boston Medical and Surgical Journal; Boston Journal of Chemistry; Baltimore Physician and Surgeon; Cincinnati Lancetand Observer; Cincinnati Medical News; The Clinic; Chicago Medical Journal; Detroit Review of Medicine and Pharmacy; Druggists' Circular; The Druggist; The Galaxy; Herald of Health; Indiana Journal of Medicine; Journal of Materia Medica; London Lancet; Medical Examiner; Medical Record; Medical News and Library; Medical and Surgical Reporter; Medical Times; Nashville Journal of Medicine and Surgery; Pacific Medical and Surgical Journal; Popular Science Monthly; Physician and Pharmacist; St. Louis Medical and Surgical Journal; Virginia Medical Monthly; Western Lancet.

